# 17-AAG Induces Cytoplasmic α-Synuclein Aggregate Clearance by Induction of Autophagy

**DOI:** 10.1371/journal.pone.0008753

**Published:** 2010-01-18

**Authors:** Michael Riedel, Olaf Goldbaum, Lisa Schwarz, Sebastian Schmitt, Christiane Richter-Landsberg

**Affiliations:** Department of Biology, Molecular Neurobiology, University of Oldenburg, Oldenburg, Germany; University of Nebraska, United States of America

## Abstract

**Background:**

The accumulation and aggregation of α-synuclein in nerve cells and glia are characteristic features of a number of neurodegenerative diseases termed synucleinopathies. α-Synuclein is a highly soluble protein which in a nucleation dependent process is capable of self-aggregation. The causes underlying aggregate formation are not yet understood, impairment of the proteolytic degradation systems might be involved.

**Methodology/Principal Findings:**

In the present study the possible aggregate clearing effects of the geldanamycin analogue 17-AAG (17-(Allylamino)-17-demethoxygeldanamycin) was investigated. Towards this, an oligodendroglial cell line (OLN-93 cells), stably expressing human α-synuclein (A53T mutation) was used. In these cells small punctate aggregates, not staining with thioflavine S, representing prefibrillary aggregates, occur characteristically. Our data demonstrate that 17-AAG attenuated the formation of α-synuclein aggregates by stimulating macroautophagy. By blocking the lysosomal compartment with NH_4_Cl the aggregate clearing effects of 17-AAG were abolished and α-synuclein deposits were enlarged. Analysis of LC3-II immunoreactivity, which is an indicator of autophagosome formation, further revealed that 17-AAG led to the recruitment of LC3-II and to the formation of LC3 positive puncta. This effect was also observed in cultured oligodendrocytes derived from the brains of newborn rats. Inhibition of macroautophagy by 3-methyladenine prevented 17-AAG induced occurrence of LC3 positive puncta as well as the removal of α-synuclein aggregates in OLN-A53T cells.

**Conclusions:**

Our data demonstrate for the first time that 17-AAG not only causes the upregulation of heat shock proteins, but also is an effective inducer of the autophagic pathway by which α-synuclein can be removed. Hence geldanamycin derivatives may provide a means to modulate autophagy in neural cells, thereby ameliorating pathogenic aggregate formation and protecting the cells during disease and aging.

## Introduction

In Parkinson's disease (PD) the accumulation and aggregation of α-synuclein in neurons is a characteristic feature, while α-synuclein positive glial cytoplasmic inclusions (GCIs) originating in oligodendrocytes are the histological hallmark of multiple system atrophy (MSA), a specific adult onset neurodegenerative disease with symptoms of Parkinsonism [1], [2]. These inclusions are further characterized by staining with antibodies against ubiquitin and a variety of heat shock proteins (HSPs), specifically the small HSP αB-crystallin. Various reports indicate the presence of the microtubule associated protein tau [3], [4]; for a recent review see [5]. Also, HSP90 was found to be predominantly associated in ubiquitinated inclusions of α-synucleinopathies [6]. HSPs participate in protein folding, protein translocation and transport processes. They function as molecular chaperones and guide misfolded proteins to the proteasomal machinery for ubiquitination and degradation [7].

α-Synuclein is highly soluble and a natively unfolded protein, which in a nucleation dependent process is capable of self-aggregation. This may yield potentially neurotoxic non-fibrillar oligomers or protofibrils and fibrillar aggregates with amyloid characteristics [8], [9], [10]. α-Synuclein has been demonstrated to be present in oligodendrocytes and astrocytes in normal human brain [11] and we have shown previously that in cultured rat brain oligodendrocytes α-synuclein mRNA and protein is present and downregulated during culture maturation [12], [13]. The mechanisms underlying GCI formation are rather elusive, and the causes of α-synuclein overexpression and aggregate formation are not yet understood. Impairment of the proteolytic degradation systems might contribute to pathogenic consequences [14], [15]. α-Synuclein degradation occurs by both the proteasome and autophagic pathways within lysosomes [16], [17], [18], [19], [20] The co-chaperone CHIP (carboxyl terminus of Hsp70-interacting protein) has been suggested to be involved as a molecular switch between the two degradation pathways [21]. Furthermore, extensive accumulation of α-synuclein is associated with lysosomal alterations [22]. As we have shown before, stable expression of α-synuclein or the A53T mutation of α-synuclein in OLN-t40 oligodendroglial cells [23] did not exert cytotoxic responses, but caused the formation of small punctate non-fibrillary α-synuclein aggregates which were more prominent in cells expressing the mutation [24].

In the present study we have investigated the possible aggregate-clearing effects of the geldanamycin analogue 17-AAG. 17-AAG is currently in clinical trials as an anticancer drug, specifically binds to and inhibits HSP90 [25] and triggers the activation of a heat shock response in mammalian cells [26], [27]. Our data demonstrate for the first time that 17-AAG not only causes the upregulation of HSPs, but also is an effective inducer of the autophagic pathway and thereby promotes the removal of prefibrillary α-synuclein aggregates.

## Materials and Methods

### Materials and Antibodies

Cell culture media were from Gibco/BRL (Grand Island, NY). MG-132 (carbobenzoxy-L-leucyl-L-leucyl-L-leucinal) and proteolytic substrate II (Z-Leu-Leu-Glu-AMC) were purchased from Merck KGaA (Darmstadt, Germany). Rapamycin was purchased from Santa Cruz (Heidelberg, Germany). Ammoniumchloride, 3-Methyladenine (3-MA), Chloroquine, ATP and neutral red were from Sigma (Seelze, Germany). MTT (1-(4,5-Dimethylthiazol-2-yl)-3,5-diphenylformazan) was from USB Corporation (Cleveland, OH, USA). 17-(Allylamino)-17-demethoxygeldanamycin (17-AAG) was from A.G. Scientific, Inc. (San Diego, CA, USA). For Western blot analysis the following antibodies were used, the working dilutions are given in brackets. Rabbit polyclonal antibody (PAb) anti-α-synuclein (SNL-4, 1∶1000) was from Dr. Viginia Lee, Philadelphia, USA. Rabbit PAb anti-myelin basic protein (MBP) antibody (1∶1000) was a generous gift of Dr. A. McMorris (Wistar Institute, Philadelphia, PA, USA).

Monoclonal antibody (MAb) anti-α-tubulin (1∶1000) was from Sigma (Seelze, Germany). MAb anti-LC3 (1∶250) was from Nanotools (Teningen, Germany). MAb anti-αB-crystallin (1∶1000), PAb anti-HSP32/HO-1 (1∶1000), MAb anti-HSP70 (1∶1000) and MAb anti-HSP90 (1∶1000) were from StressGen (Ann Arbor, MI, USA). HRP-conjugated anti-mouse IgG was from Amersham (Freiburg, Germany) and anti-rabbit IgG from Biorad (Munich, Germany).

### Cell Culture and Transfection

Cells were kept in DMEM supplemented with 10% heat-inactivated fetal calf serum, 2 mM Glutamine, 50 U/ml penicillin and 50 µg/ml streptomycin [28]. OLN-93 cells were cotransfected with Tau40 cDNA and pcDNA3.1 containing the neomycin gene, by using the calcium phosphate precipitation method [23]. After selection in DMEM containing 1.0 mg/ml G418, the cells were screened for tau expression by Western blot and indirect immunofluorescence. A stable cell line was established, designated OLN-t40, which was then infected with recombinant lentiviral vector (Invitrogen, Grand Island, NY) to stably express human wild type α-synuclein or mutant human A53T α-synuclein (OLN-A53T).

Oligodendrocytes were prepared as described previously [13], [29]. Briefly, primary cultures of glial cells were prepared from the brains of 1–2-day-old Wistar rats and oligodendrocytes were mechanically removed from the flasks after 6–8 days. Precursor cells were replated on poly-L-lysine (PLL)-coated culture dishes (2.7×10^6^ cells/10 cm dish) and kept for 5–7 days in serum-free DMEM to which insulin (5 µg/ml), transferrin (5 µg/ml), and sodium selenite (5 ng/ml) (Roche Diagnostics, Mannheim, Germany) was added. These cultures contain a highly enriched population of differentiated oligodendrocytes with a mature morphology [13].

### Heat Shock Treatment

Culture dishes were sealed with Parafilm and immersed for 30 min in a water bath at 44°C, as described [29]. Thereafter, the cells were put into the incubator for 24 h of recovery. Control cells were sealed for 30 min but remained in the incubator.

### Immunoblot Analysis

Cellular monolayers of control and treated cells were washed with PBS once, scraped off in sample buffer containing 1% SDS and boiled for 10 min. Protein contents in the samples were determined according to [30]. For immunoblotting, total cellular extracts (5–20 µg protein per lane) were separated by one-dimensional SDS-PAGE using 7.5% or 12.5% polyacrylamide gels and transferred to nitrocellulose membranes (Whatman, Dassel, Germany; 0.2 µm). For LC3 transfer PVDF blotting membrane (Whatman, Dassel, Germany; 0.2 µm) was used. The blots were saturated with TBS (20 mM Tris, 136.8 mM NaCl, pH 7.5) containing 5% dry milk and incubated with the individual antibodies overnight at 4°C. After washing with TBS-T (TBS with 0.1% v/v Tween 20), incubation with HRP-conjugated anti-mouse (1∶3000) or anti-rabbit IgG (1∶3000) was carried out for 1 h at RT. After washing with TBS-T, blots were visualized by the enhanced chemiluminescence (ECL) procedure as described by the manufacturer (Amersham, Braunschweig). All experiments were carried out at least 3 times with similar results.

### Immunocytochemistry

Cells were cultured on poly-L-lysine-coated glass coverslips (3×10^4^ cells per 35 mm dish) in DMEM/10% FCS and then subjected to ammoniumchloride, 17-AAG, 3-MA or rapamycin as indicated. After washing with PBS, cells were fixed with 100% icecold methanol for 7 min (for LC3) without further permeabilization or with 3% paraformaldehyde and permeabilized with 0.1% Triton for 15 min. After blocking of unspecific binding sites with 5% bovine serum albumin in PBS cells were washed three times and incubated overnight at 4°C with the following antibodies, the working dilutions are given in brackets: rabbit pAb anti-α-synuclein (SNL-4; 1∶400), mouse mAb α-tubulin (1∶400) or mouse mAb anti-LC3 (1∶100). After washing with PBS, cells were incubated for 1 h with Texas Red-conjugated (1∶100) and FITC-conjugated (1∶100) secondary antibodies (Jackson ImmunoResearch, West Grove, PA, USA), washed with PBS and mounted. Nuclei were stained by 4′,6-diamidino-2-phenylindole (DAPI) (1.5 µg/ml) included in the mounting medium (Vectashield; Vector Laboratories, Burlingame, CA, USA). Fluorescent labeling was studied using a Zeiss epifluorescence microscope (Oberkochen, Germany) equipped with a digital camera using a plan-neofluar objective (100x) or a Leica TCS SL confocal laser scanning microscope (Wetzlar, Germany).

### Proteasome Activity Assays

Proteasome activity was determined using fluorescence assays. Post-glutamyl-peptidase-hydrolase activity of the proteasome was assayed by fluorometric measurement of the release of 7-amido-4-methylcoumarin from the synthetic substrate Z-Leu-Leu-Glu-AMC (proteasome substrate II, S2). Proteasome activity was determined in cell lysates treated with 17-AAG, which assesses if 17-AAG directly binds to the proteasome, and also in cell extracts derived from live cells treated with 17-AAG, which assesses the influence of 17-AAG on proteasomal activity in live cells.

Measurement of proteasome activity in cytoplasmic lysates was carried out as described by Kumar et al. [31]. Briefly, OLN-A53T cells were kept as described, harvested in PBS, centrifuged, resuspended in HEPES buffer (5 mM HEPES, 1 mM EDTA, pH 7.5) and sonicated. After centrifugation at 14.000 rpm at 4°C for 15 min the supernatant was used to assay proteasome activity. For each sample, protein concentration was determined by the bicinchoninic acid method (Pierce, Rockford, IL, USA) using bovine serum albumin as a standard. For each sample 15 µg cellular extract was added to 5 wells of a 96-well plate containing 250 µl of HEPES buffer (20 mM HEPES, 0.5 mM EDTA, 0.035% SDS, pH 8) each. MG-132 (1 µM) or 17-AAG (50 nM) were added to the cellular extract and incubated for 60 min. After adding 5 µl proteasome substrate II, the contents were incubated for additional 30 min at 37°C. Finally the hydrolysis of the substrate was measured by a fluorometer at 380 nm excitation wavelength and 440 nm emission wavelength.

Proteasome activity in cell lysates prepared after treatment of live cells with 17-AAG was determined as described by Keller et al. [32]. OLN-A53T were incubated with 17-AAG (50 nM, 24 h), and harvested in ice cold proteolysis assay buffer containing 10 mM Tris-HCl (pH 7.2), 0.035% SDS, 5 mM MgCl_2_ and 5 mM ATP and sonicated. Protein concentrations of the resulting lysates were determined by the bicinchoninic acid method (Pierce, Rockford, IL) using bovine serum albumin as a standard. Aliquots of 350 µl each, with a protein concentration of 1 µg/µl, were incubated with 3.5 µl of proteasome substrate II (5 mM) at 37°C. Fluorescence was determined after 30 min at 380 nm excitation and 440 nm emission in a fluorescent microplate reader (FluoroCount, Packard). Proteasomal activity was determined as an increase in fluorescence of the reaction products. Each experiment was repeated 3 times involving 5 samples per group.

### Cytotoxicity - Assays

To assess the cytotoxic potential of the compounds, the MTT- and Neutralred assays were carried out. Briefly, OLN cells were plated on PLL-coated 96-microwell cell culture plates (3×10^3^ cells/well) and grown in DMEM/10% FCS. Cells were stressed for 24 h with indicated concentrations and assays were performed.

*MTT-Assay:* Ten microliters of MTT solution (5 mg/ml in PBS) was added to the wells containing 100 µl medium and plates were incubated for 4 h. Thereafter, 100 µl of a solubilization solution (10% sodium dodecyl sulfate in 0.01 mol/l HCl) was added and incubated overnight to dissolve the formazan salt. Quantification was then carried out with a microplate reader (Biorad, Munich, Germany) at 595 nm, using a 655 nm filter as a reference. Data are expressed as percentage of the untreated controls, and values represent the means ± SD of sixteen microwells each of two independent experiments (*n* = 32).

*Neutral Red-Assay:* For neutral red assay [33] cells were washed with PBS and incubated for 3 h in medium containing neutral red (0.005%). Cells were washed with PBS and dye was extracted with 100 µl of a mixture of 1% acetic acid and 50% methanol. Quantification was then carried out with a microplate reader (Biorad, Munich, Germany) at 540 nm. Data are expressed as percentage of the untreated controls, and values represent the means ± SD of sixteen microwells each of two independent experiments (*n* = 32).

### Statistics

Results are expressed as mean ± SEM from at least three independent experiments or as indicated. Multiple group comparisons were performed using one-way analysis of variance (ANOVA) and Fisher's least significant difference (LSD). Values of P ≤0.01 were defined as statistically significant.

## Results

The present investigation was carried out with oligodendroglial OLN-93 cells, an oligodendroglial cell line established from primary glial cultures derived from the brains of newborn rats [28]. These cells were stably engineered to express the longest human isoform of tau [23] and wild-type α-synuclein or the A53T mutation. OLN-93 cells were more easily transfectable with α-synuclein or α-synuclein mutations when tau was present, indicating a protective role of tau. As we have shown before stable transfection of these cells with α-synuclein or mutant α-synuclein A53T was not cytotoxic, but caused the appearance of small punctated α-synuclein aggregates, which were more prominent in the cell line expressing the α-synuclein mutation, namely OLN-A53T cells [24], which was used in the following studies.

### 17-AAG Causes the Clearance of Small α-Synuclein Aggregates and Leads to the Induction of Heat Shock Proteins

**Figure 1:**
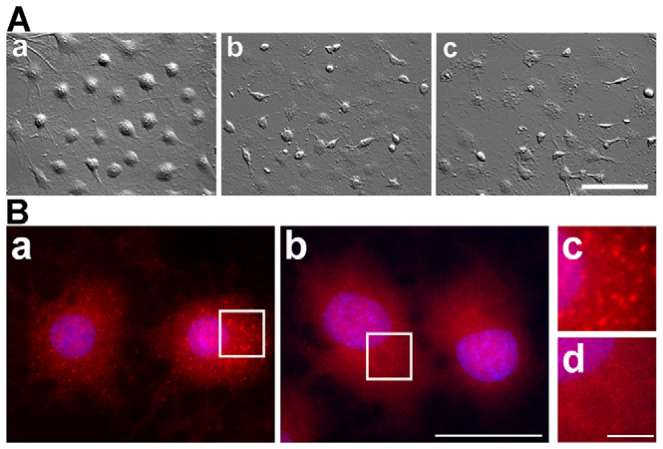
Small punctated α-synuclein aggregates are removed by 17-AAG. (A) Hoffman modulation contrast images of OLN-A53T cells are shown. *Scale bar*, 75 µm. Cells were either untreated (a) or treated with 50 nM 17-AAG for 24 h (b) or 48 h (c). (B) Cells were subjected to indirect immunofluorescence using antibodies against α-syn (SNL-4, red). Nuclei were stained with DAPI. Cells were either untreated (a) or treated with 50 nM 17-AAG for 24 h (b). In c and d, enlargements of the respective regions indicated in a and b are shown. *Scale bars*, 20 µm (a, b), 2.5 µm (c, d).

**Figure 2:**
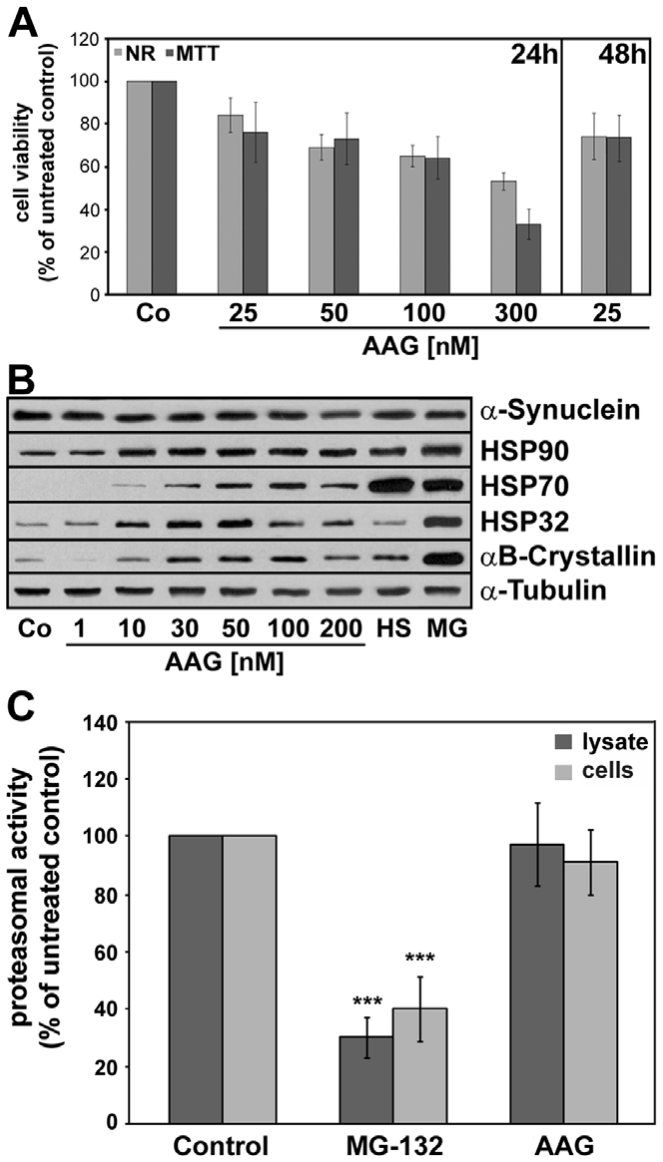
17-AAG induces a heat shock response in OLN-A53T cells and does not impair proteasomal activity. (A) Determination of cytotoxic potential. Cells were exposed to different concentrations of 17-AAG as indicated. After 24 h, neutral red and MTT assays were carried out. Values represent the mean ± SEM of 16 microwells each of two independent experiments (n = 32). (B) Immunoblot analysis of heat shock protein induction. Cells were treated with 17-AAG (1–200 nM, 24 h), or subjected to heat shock (HS: 44°C, 30 min, 24 h recovery) or to MG-132 (MG: 1 µM, 24 h). Cell lysates were prepared and immunoblot analysis was carried out with antibodies against the individual proteins as indicated on the right. Co, untreated control. (C) Proteasomal activity was determined in cell lysates treated with 17-AAG (lysate) and in cell lysates prepared from 17-AAG treated live cells (cells). Cytoplasmic lysates were incubated with the proteasomal inhibitor MG-132 (1 µM, 60 min) as a positive control, or 17-AAG (50 nM, 60 min). Cells were treated with MG-132 (1 µM, 24 h) and 17-AAG (50 nM, 24 h). The post-glutamyl-peptidase-hydrolase activity was determined using fluorogenic substrate Z-Leu-Leu-Glu-AMC (see Materials and Methods). The cleavage of the substrate is inhibited by MG-132 but not by 17-AAG. Data are expressed as percent of the untreated control and show the mean ± SEM from 3 independent experiments. Statistical evaluation was carried out by ANOVA/Fisher's LSD: ***p≤0.01 for MG-132 versus control.

The small punctated α-synuclein aggregates in these cells do not stain with thioflavine S and thus represent a prefibrillary species [24]. Tau is not a component of the prefibrillary species. Fig. 1 demonstrates that incubation of the cells with 17-AAG (50 nM) for 24 h caused morphological changes and the clearance of these aggregates. Cells appeared more flattened and partly damaged. To further determine the cytotoxic potential of 17-AAG in OLN-A53T cells, cells were treated with 17-AAG at increasing concentrations for 24 h and cell survival was analyzed. Half maximal cytotoxicity, as determined by neutral red acid uptake or MTT assay, was observed at a concentration of approximately 300 nM, and at a concentration of 25–50 nM about 20 per cent of the cells were affected (Fig. 2A). Geldanamycin was similarly cytotoxic (not shown). After 48 h of treatment with 17-AAG (25–50 nM) no further damage was observable (Fig. 1A,c and 2A).

Geldanamycin and its analogue 17-AAG are inhibitors of HSP90 [25], have been demonstrated to activate a heat shock response [26], [27], and possibly act through the increased expression of molecular chaperones, in particular through HSP70. To test if these compounds lead to the induction of HSPs in the present cell culture system, immunoblot analysis was carried out using a panel of antibodies against HSPs (Fig. 2B). The data demonstrate that 17-AAG in a concentration dependent manner, within 24 h caused the upregulation of several HSPs, including HSP90, HSP70, HSP32 and αB-crystallin.

The amount of ubiquitinated proteins was not changed by 17-AAG (not shown). However, specifically the induction of HSP70, which has been connected to the inhibition of α-synuclein fibril formation, aggregation and toxicity [34], [35], was observable but occurred to a much lower extent than after a heat shock (44°C, 30 min, 24 h recovery) or after proteasomal inhibition by MG-132 (1 µM, 24 h) (Fig. 2B). Hence the aggregate clearing potential of 17-AAG might be causally related to other mechanisms, such as induction of the proteolytic capacity of the cells.

### Aggregate Clearance by 17-AAG Involves Lysosomal Degradation Pathways

**Figure 3:**
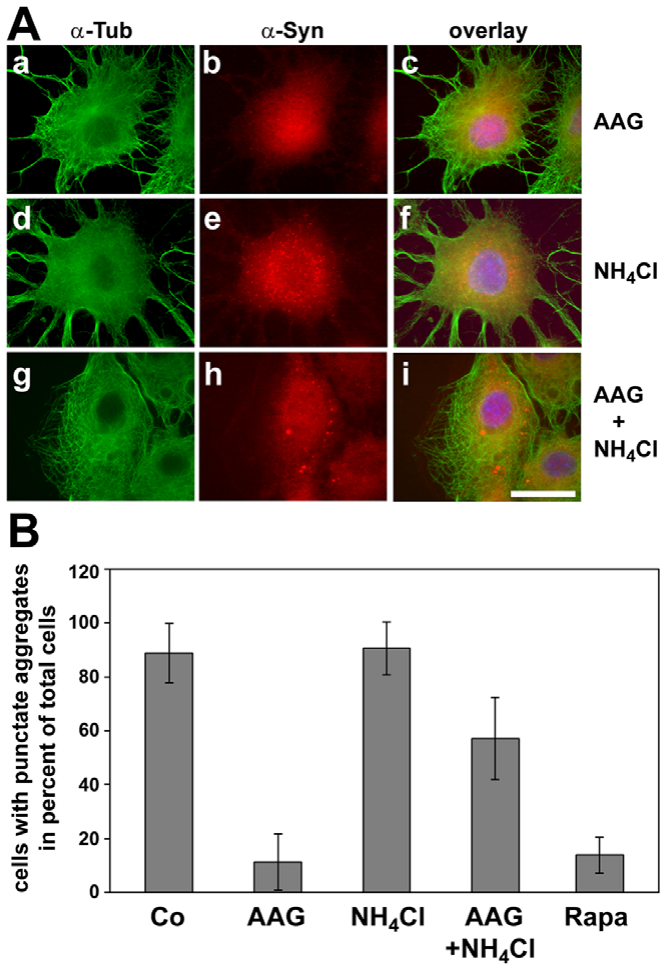
17-AAG induced clearance of α-synuclein aggregates is prevented by lysosomal inhibition. (A) Cells were treated either with 50 nM 17-AAG (AAG; a–c), or with 50 mM NH_4_Cl (d–f) for 24 h or with a combination of both (g–i). Cells were fixed with paraformaldehyde and indirect immunofluorescence staining was carried out using antibodies against α-tubulin (a, d, g; green) and α-synuclein (b, e, h; red). Overlay with DAPI (c, f, i). *Scale bar*, 20 µm. Note that in cells treated with NH_4_Cl aggregates remain or are even enlarged. (B) Quantitative evaluation of the percentage of cells expressing puntacte α-synuclein aggregates. At least 350 cells on four cover slips each of two independent experiments were counted. Data are expressed as per cent of total cells +/− SD. Experimental conditions were as in (A). Additionally, cells treated with rapamycin (Rapa, 5 µM, 24 h) were counted.

First we tested if 17-AAG enhances proteasomal activity in OLN-A53T cells. Cell lysates were prepared and proteasome activities were determined as described by [31], [32]. As indicated in Fig. 2C, 17-AAG (50 nM) did not enhance or impair proteasomal activity, while the proteasome inhibitor MG-132 (1 µM) effectively reduced proteasome activity by about 60–70 per cent. Furthermore, α-synuclein aggregate formation was not promoted by MG-132 (not shown). To assess whether the aggregates were removed by 17-AAG-stimulated lysosomal degradation, cells were treated with the lysosomal inhibitor NH_4_Cl (50 mM) for 24 h either alone or in combination with 17-AAG (50 nM). In the presence of NH_4_Cl, the aggregates remained and were enlarged (Fig. 3A). This was also observed when cells were incubated with 17-AAG and the lysosomal inhibitor chloroquine simultaneously (data not shown). Quantitative evaluation, as depicted in Fig. 3B, revealed that the percentage of cells containing punctated α-synuclein aggregates in control cells and cells treated with NH_4_Cl was about 90%, while in cells treated with 17-AAG or rapamycin, an effective inducer of autophagy [36], only 10–15% carried small aggregates (Fig. 3B). In cultures treated with 17-AAG and NH_4_Cl simultaneously, about 60% of the cells contained small aggregates. These results indicate that 17-AAG promotes the clearance of the small α-synuclein accumulations via lysosomal pathways.

**Figure 4:**
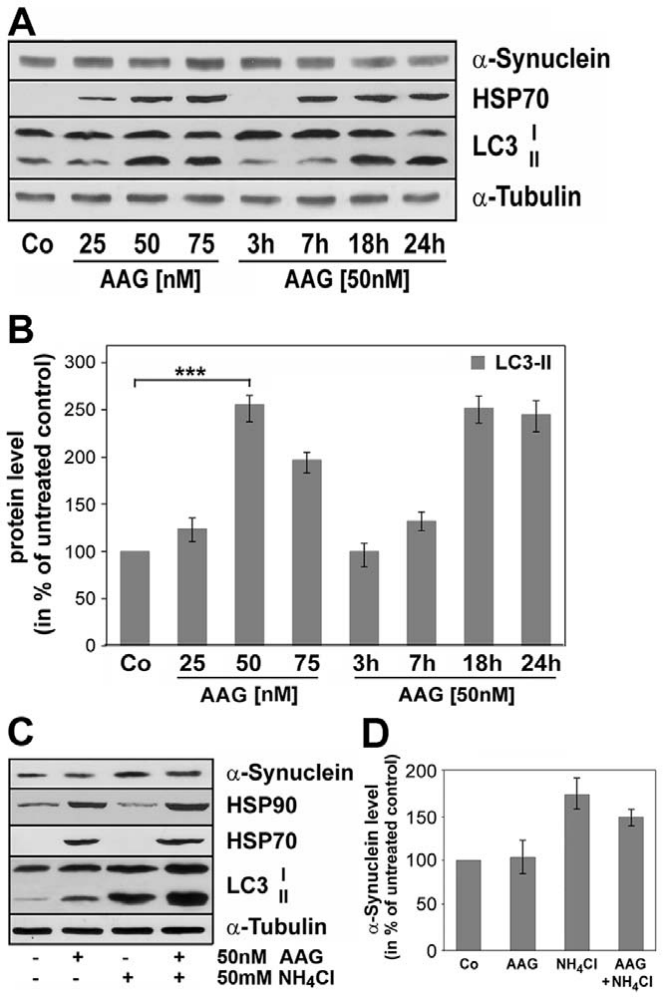
17-AAG leads to induction of macroautophagy in a time and concentration dependent manner in OLN-A53T cells. (A) Immunoblot analysis of LC3. Cell lysates were prepared from OLN-A53T cells treated for 24 h with different concentrations of 17-AAG (25–75 nM) and from cells treated with 50 nM 17-AAG for different times (3 h–24 h). Immunoblot analysis was carried out with antibodies against the individual proteins as indicated on the right. Co, untreated control. 17-AAG causes the recruitment of LC3-II, an indicator of macroautophagy. A representative blot of three independent experiments is shown. (B) Quantitative evaluation of LC3-II protein level was carried out by densitometric scanning of the blots. Results show the mean ± SEM from three independent experiments. LC3-II levels were normalized to α-tubulin and the total amount of control was set to 100%. Statistical evaluation was carried out by ANOVA/Fisher's LSD: ***P±0.01 for 50 nM 17AAG (24 h) versus control (Co). (C) Lysosomal inhibition augments LC3-II levels, indicating the disturbance of the autophagic flux. Cells were treated for 24 h with 17-AAG (50 nM), or NH_4_Cl (50 mM) or with a combination of both, as indicated. Cell lysates were prepared and immunoblot analysis was carried out with antibodies against the individual proteins as indicated on the right. Co, untreated control. (D) Quantitative evaluation of the α-synuclein levels. Experimental conditions as in (C). Data are the means of two independent experiments +/− SD.

Hence we probed for LC3, a specific marker for autophagosomes, to test if autophagic activity was induced by 17-AAG. During autophagosome formation endogenous LC3 is processed to LC3-I, an 18 kDa cytosolic isoform, which is converted to LC3-II. The latter is a membrane-bound 16 kDa isoform which associates with the autophagosomal membranes and its amount as compared to tubulin or actin correlates with the number of autophagosomes [37], [38]. Cells were incubated for 24 h with increasing concentrations of 17-AAG (25–75 nM) or with 50 nM 17-AAG for 3–24 h, cell lysates were prepared and subjected to immunoblot analysis. Fig. 4A demonstrates that 17-AAG in a time- and concentration dependent manner markedly increased the level of LC3-II. Quantitative evaluation indicates that this effect is maximal after 18–24 h at a concentration of 50 nM (Fig. 4B).

Since LC3-II itself is degraded by autophagy, we compared LC3-II levels in the absence and presence of the lysosomal inhibitor NH_4_Cl. Immunoblot analysis revealed that when cells were incubated with 17-AAG (50 nM) in combination with NH_4_Cl (50 mM) for 24, the level of LC3-II was further augmented in comparison to the treatment with 17-AAG alone (Fig. 4C), pointing to an enhancement of the autophagic flux by 17-AAG [38]. Neither NH_4_Cl nor chloroquine (25 µM; 24 h) alone caused the upregulation of HSP70 (Fig. 4C) or of any other HSPs tested. Quantitative evaluation of the immunoblots indicated that in the presence of NH_4_Cl the amount of α-synuclein was enhanced supporting the notion that the lysosomal pathway is involved in its degradation (Fig. 4D).

**Figure 5:**
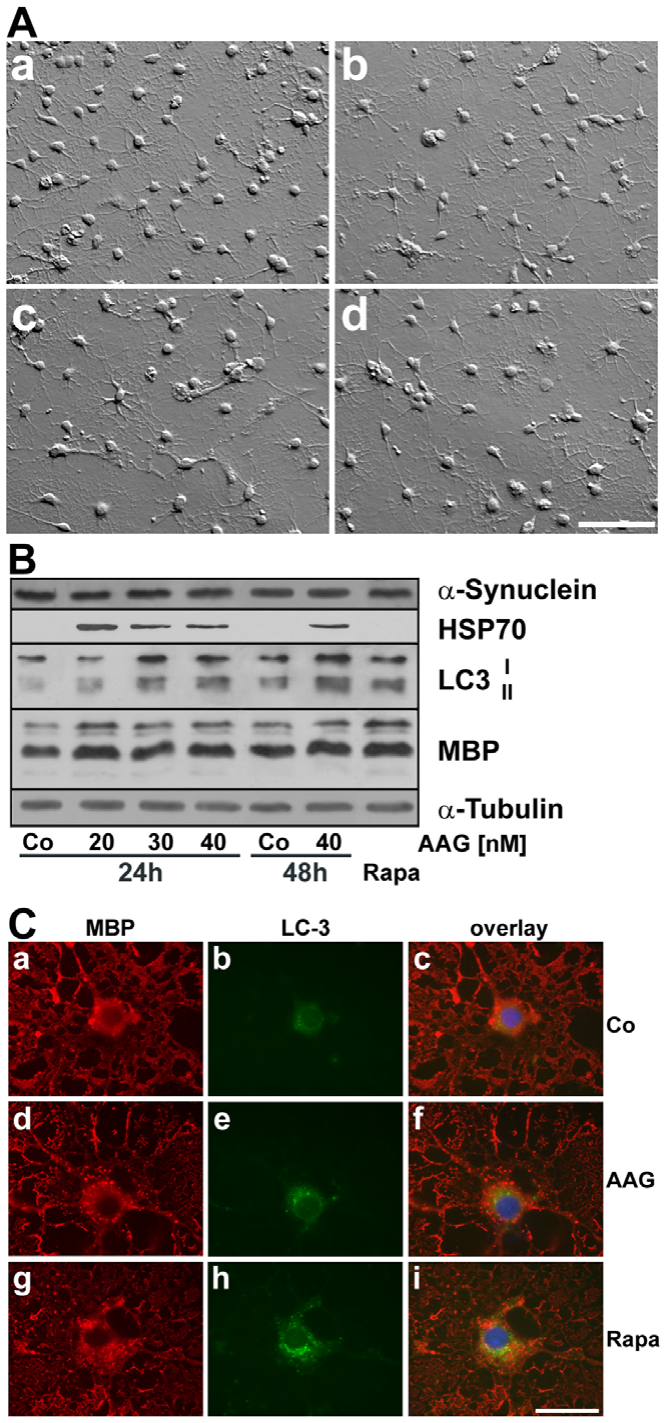
17-AAG leads to the induction of macroautophagy in cultured rat brain oligodendrocytes. (A) Hoffman modulation contrast images are shown. Oligodendrocytes (5 div) were prepared from the brains of newborn rats and subjected to 50 nM 17-AAG for 24 h (c) or 48 h (d) or to 5 µM rapamycin for 24 h (b). In (a) the untreated control is shown. Scale bar: 50 µm. (B) Immunoblot analysis of LC3. Cell lysates were prepared from oligodendrocytes (5 div) treated for 24 h or 48 h with different concentrations of 17-AAG (20–40 nM) or with rapamycin (Rapa: 5 µM, 24 h). Immunoblot analysis was carried out with antibodies against the individual proteins as indicated on the right. Co, untreated control. (C) Oligodendrocytes (5 div) were either untreated (Co, a–c), or treated for 24 h with 40 nM 17AAG (AAG, d–f), or with 5 µM rapamycin (Rapa, g–i), and then subjected to indirect immunofluorescence staining using antibodies against myelin basic proteins (MBP, a,d,g; red) and LC3 (b,e,h; green). In c,f,i the overlays with DAPI staining are shown. *Scale bar*, 25 µm.

To assess if the stimulatory effects of 17-AAG on macroautophagy is not restricted to the oligodendroglial clonal cell line used in this study, primary cultures of rat brain oligodendrocytes were prepared and subjected to 17-AAG. Oligodendrocytes treated with 17-AAG (24 h and 48 h, 40 nM) remained morphologically intact and displayed an arborized morphology (Fig. 5A). Immunoblot analysis further indicated that 17-AAG (20–40 nM; 24–48 h) in oligodendrocytes increased the levels of LC3-II (Fig. 5B). Also rapamycin (5 µM; 24 h) caused an increase in LC3-II (Fig. 5B). Indirect immunofluorescence corroborated this finding, demonstrating the accumulation of LC3-positive puncta in the cell somata similarly as observed after treatment with the macroautophagy inducer rapamycin (Fig. 5C). Similarly to primary cultures of oligodendrocytes, 17-AAG caused a marked increase in the level of LC3-II in OLN cells stably expressing α-synuclein in the absence of tau (data not shown). However, both cell culture systems do not express prefibrillary α-synuclein aggregates under normal growth conditions, hence, OLN-A53T cells were used to further characterize the effects of 17-AAG on α-synuclein clearance.

**Figure 6:**
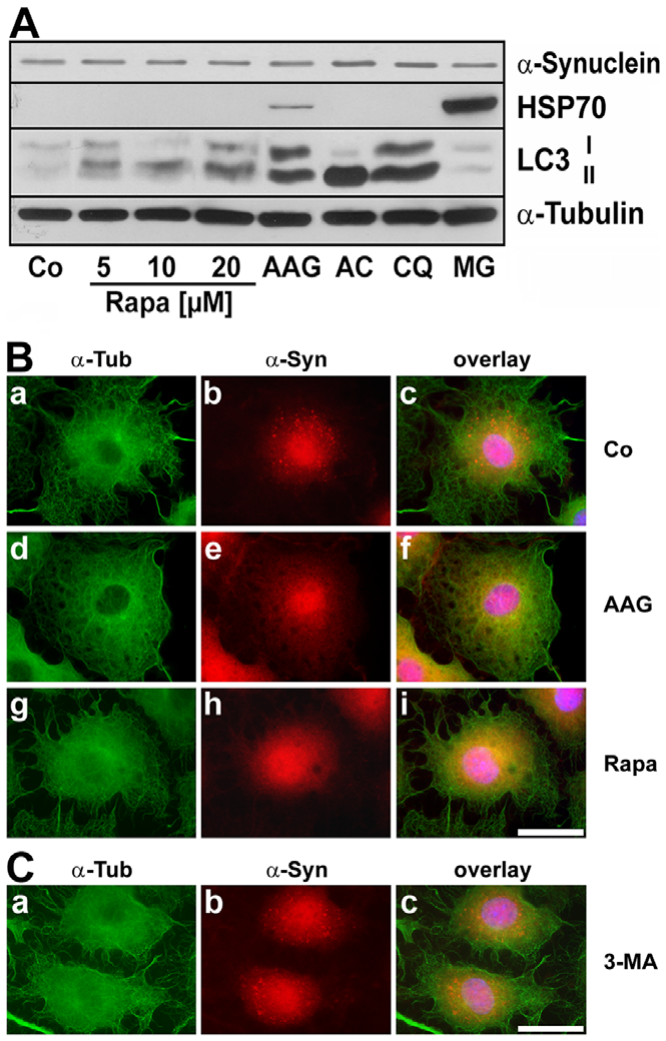
Autophagy induction by rapamycin causes aggregate clearance similarly to 17-AAG. (A) Immunoblot analysis of LC3 and HSP70. Cells were treated for 24 h with rapamycin (Rapa, 5–20 µM), 17-AAG (AAG, 50 nM), ammonium chloride (AC, 50 mM), chloroquine (CQ, 25 µM) or MG-132 (MG, 1 µM) and subjected to immunoblot analysis using antibodies against HSP70, LC3 and α-tubulin, as indicated on the right. Co, untreated control. (B) OLN-A53T cells were untreated (Co, a–c), or treated for 24 h with 50 nM 17-AAG (AAG, d–f) or with 20 µM rapamycin (Rapa, g–i) and then subjected to indirect immunofluorescence staining using antibodies against α-tubulin (a,d,g; green) and α-synuclein (SNL-4, b,e,h; red). In (c,f,i) the overlays with DAPI staining are shown. *Scale bar*, 20 µm. (C) Inhibition of macroautophagy by 3-methyladenine. OLN-A53T cells were treated for 24 h with 50 nM 17AAG in combination with 3-MA (10 mM) and then subjected to indirect immunofluorescence staining using antibodies against α-tubulin (a) and α-synuclein (SNL-4, b). In (c) the overlay with DAPI staining is shown. Note that the clearance of punctated α-synuclein aggregates is inhibited by 3-MA. *Scale bar*, 20 µm.

To evaluate the efficiency of 17-AAG in inducing the autophagic pathway, we compared its effect to rapamycin in OLN-A53T cells. Analysis of cell lysates by immunoblot procedure shows that rapamycin (5–20 µM; 24 h) caused an enhancement of LC3-II levels, however under these conditions not as efficiently as 17-AAG (50 nM; 24 h) (Fig. 6A). While proteasomal inhibition by MG-132 (1 µM; 24 h) did not affect LC3, both lysososomal inhibitors chloroquine (25 µM; 24 h) and NH_4_Cl (50 mM; 24 h) caused increased LC3-II levels by inhibiting its degradation (Fig. 6A). Furthermore, as indicated by indirect immunofluorescence staining, rapamycin similarly to 17-AAG promoted the clearance of the small α-synuclein aggregates (Fig. 6B, see also Fig. 3B). The effects of rapamycin were not accompanied by an induction of HSP70 (Fig. 6A).

### 3-Methyladenine Inhibits 17-AAG Induced Autophagy and LC3 Puncta Formation

**Figure 7:**
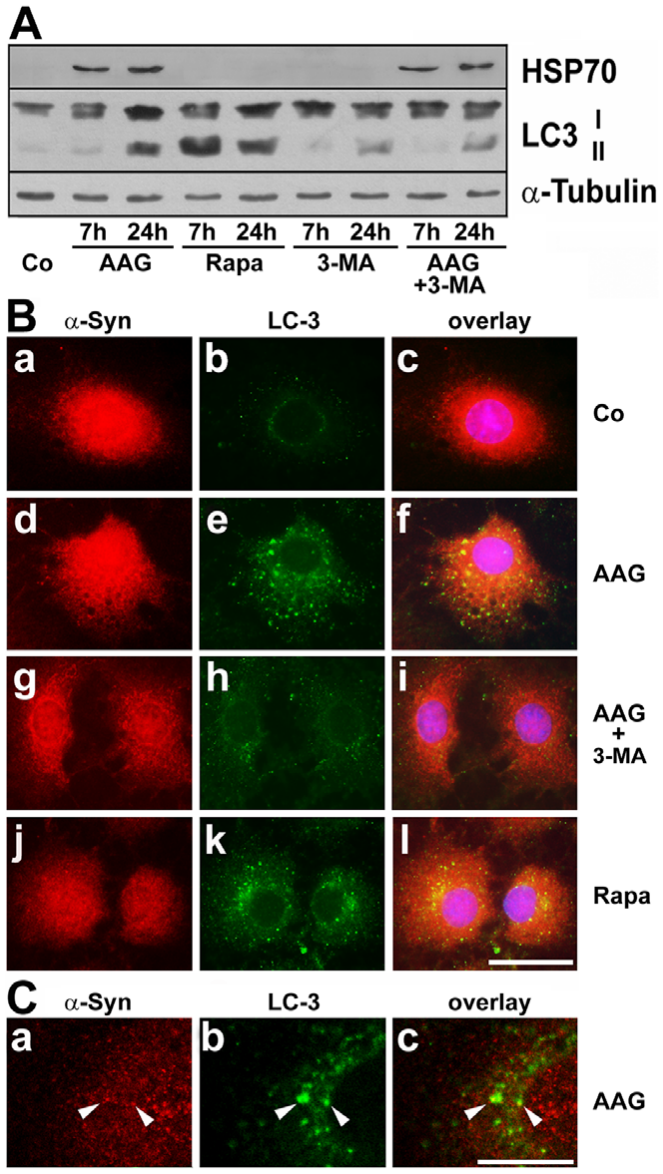
17-AAG and rapamycin promote the accumulation of LC3-II and the formation of LC3 positive puncta which is inhibited by 3-MA. (A) Immunoblot analysis of LC3 and HSP70. Cells were treated with 17-AAG (AAG, 50 nM), rapamycin (20 µM), or 3-methyladenine (3-MA, 10 mM) or with a combination of 17-AAG and 3-MA for 7 h and 24 h, respectively. Cell lysates were prepared and immunoblot analysis was carried out with antibodies against the individual proteins as indicated on the right. Co, untreated control. (B) OLN-A53T cells were either untreated (Co, a–c), or treated for 24 h with 50 nM 17AAG (AAG, d–f), or with a combination of 50 nM 17AAG and 10 mM 3-methyladenine (3-MA, g–i), or with 20 µM rapamycin (Rapa, j–l), and then subjected to indirect immunofluorescence staining using antibodies against α-synuclein (SNL-4, a,d,g,j; red) and LC3 (b,e,h,k, green). In (c,f,i,l) the overlays with DAPI staining are shown. *Scale bar*, 25 µm. (C) Confocal images of cells treated with 17-AAG as in (B, d–f) are shown. Arrow heads indicate colocalization of LC3 staining with α-synuclein. *Scale bar*, 5 µm.

The contribution of macroautophagy to the degradation of α-synuclein in OLN-A53T cells was further confirmed by using the selective inhibitor of macroautophagy, 3-methyladenine (3-MA). In cells incubated in the presence of 17-AAG (50 nM) and 3-MA (10 mM) simultaneously for 24 h, α-synuclein positive aggregates remained to be present throughout the cytoplasm, and thus the aggregate clearing effect of 17-AAG was abolished (Fig. 6C). Immunoblot analysis of cell extracts depicted that application of 3-MA (10 mM) alone or in combination with 17-AAG (50 nM; 7–24 h) prevented (after 7 h) or reduced (after 24 h) the formation of LC3-II, while the induction of HSP70 was not affected (Fig. 7A). Additionally as demonstrated by indirect immunofluorecence staining, in cells treated for 24 h with 17-AAG (50 nM) or rapamycin (20 µM) alone, LC3 positive puncta had been formed abundantly and were seen throughout the cytoplasma (Fig. 7B). In contrast thereto in control cells and cells treated for 24 h with 3-MA (10 mM) and 17-AAG (50 nM) in combination, LC3 immunoreactivity was rarely seen (Fig. 7B). Also, confocal microscopy indicates that in cells after treatment with 17-AAG, α-synuclein immunoreactivity occasionally was detectable in close proximity or in colocalization with LC3-positive vesicles (Fig. 7C).

## Discussion

α-Synuclein is the major building block of Lewy bodies in PD and glial cytoplasmic inclusions in MSA [39]. Abnormal deposition of α-synuclein has been linked to the pathogenesis of neurodegenerative diseases, and missense mutations of the human gene, such as A53T, increase the probability of aggregate formation (for review see, [40], [41]. It has been hypothesized that the accumulation of prefibrillary oligomers, which provide the intermediates for fibrillary aggregates or inclusion bodies, are the toxic species and cause neurodegeneration [8], [40], [42]. Cells are capable of clearing oligomeric α-synuclein intermediates, and lysosomal pathways have been suggested to be mostly responsible for clearance of oligomers but not for fibrillar inclusion bodies [18]. Thus, stimulation of lysosomal pathways may be an effective therapeutic approach to prevent α-synuclein oligomer toxicity and accumulation.

Autophagy is a lysosomal pathway for degrading organelles and long-lived proteins. The three main types of autophagy are chaperone mediated autophagy (CMA), microautophagy and macroautophagy [15]. CMA involves the translocation of cytosolic proteins with a specific pentapeptide motif across the lysosomal membrane and this process requires the action of a number of cytosolic and lysosomal chaperones. In microautophagy small cytoplasmic contents are introduced into the lysosomes in a process which has been mainly characterized in yeast. Macroautophagy, often referred to only as autophagy, is a pathway by which organelles and parts of cytoplasm containing proteins are sequestered into a vesicle, termed autophagosome. After fusion of the autophagosome with the lysosome the contents are degraded [15], [43]. An equilibrium exists between autophagosome formation and lysosomal clearance, which has been termed autophagic flux [44], [45]. Autophagy can function as a cytoprotective response and is particularly crucial in the aging brain and during neurodegeneration [46]. α-Synuclein can be degraded either by the proteasome or by autophagy. Both macroautophagy and CMA have been reported to contribute to α-synuclein degradation [17], [20], [47], [48], however the clearance of mutant α-synuclein by CMA seems to be impaired [19].

In the present cell culture system, the stable expression of α-synuclein or the A53T mutated form leads to the accumulation of small punctate aggregates throughout the cytoplasm, which are more abundant in cells expressing the A53T mutation, but do not exert cytotoxic effects per se. These aggregates do not stain with thioflavine S [24] and thus represent non-fibrillar inclusions which might precede and are a requirement for the formation of fibrillary deposits, as has been described in COS-7 cells transiently transfected with α-synuclein [42]. Our study demonstrates that the geldanamycin analogue 17-AAG attenuates the formation of these small aggregates and that lysosomal and not proteasomal pathways are involved. By blocking the lysosomal compartment with NH_4_Cl or chloroquine, the aggregate clearing effects of 17-AAG were diminished and α-synuclein deposits were even enlarged, while on the other hand inhibition of the proteasomal activity by MG-132 did not have this effect. Analysis of LC3-II immunoreactivity, which is an indicator of autophagosome formation, further revealed that induction of macroautophagy was involved in the aggregate-clearing effects of 17-AAG. This conclusion is supported by the finding that the specific inhibitor of macroautophagy 3-MA prevented 17-AAG induced occurrence of LC3 positive puncta and removal of α-synuclein aggregates.

The capability of 17-AAG to enhance macroautophagy was further demonstrated in cultured oligodendrocytes derived from the brains of newborn rats. Under normal growth conditions and in the healthy human brain oligodendrocytes do not contain α-synuclein aggregates. However, under pathological conditions and in MSA filamentous α-synuclein inclusions are present in the oligodendroglial cytoplasm and the disease has been suggested to represent an oligodendroglia synucleinopathy [2]. In this respect, the finding that 17-AAG has the capacity to induce the autophagic pathway in oligodendrocytes might be of special interest as a therapeutic intervention.

The HSP90 inhibitor geldanamycin and its derivative 17-AAG modulate HSP90 function and facilitate the degradation of HSP90 client proteins [49], [50]. Geldanamycin has been demonstrated to activate a heat shock response and to suppress huntingtin protein aggregation in a cell culture model of Huntington's disease [26]. The stimulation of heat shock gene transcription was also attributed to its ability to protect the brain from focal ischemia [51], and geldanamycin was shown to restore a defective heat shock response in vivo [52]. Suppression of α-synuclein aggregation and toxicity by geldanamycin was observed in human H4 neuroglioma cells [27]. Furthermore, it prevented from α-synuclein toxicity in a transgenic fly model despite the continuous presence of aggregate pathology [53], [54]). These reports suggested that geldanamycin exerts its effects by upregulation of HSP70 expression. In another study, HSP70 overexpression in mice has been demonstrated to reduce α-synuclein aggregation and in vitro caused a reduction in the insolubility of α-synuclein [35]. Also, HSP70 may reduce α-synuclein fibril formation by binding preferentially to prefibrillar species [34]. On the other hand, HSP27 and not HSP70 exerted a potent protective effect against α-synuclein mediated cell death in mammalian neuronal cells [55].

Our data show that HSPs and specifically HSP70 are indeed induced by 17-AAG, but to a much lesser extent than after a heat shock or by the proteasome inhibitor MG-132, and neither rapamycin nor 3-MA modulate the heat shock response. Rapamycin did not cause the induction of HSPs, and 3-MA prevented the aggregate clearing effects of 17-AAG without interfering with HSP70 induction. This suggests that HSP70 may contribute but is not the major player in this context, and that 17-AAG-induced clearance of α-synuclein aggregates is causally related mainly to its autophagy stimulating activity. The notion that in the fly model a concentration of geldanamycin not leading to the induction of HSP70 was sufficient to protect neurons against α-synuclein toxicity [53], sustains this assumption. Hence geldanamycin and its less toxic derivatives [56] may provide a means to remove the pathological oligomeric species of α-synuclein, thereby ameliorating pathogenic aggregate formation and protecting the cells during disease and aging.
